# Fiber architecture in remodeled myocardium revealed with a quantitative diffusion CMR tractography framework and histological validation

**DOI:** 10.1186/1532-429X-14-70

**Published:** 2012-10-12

**Authors:** Choukri Mekkaoui, Shuning Huang, Howard H Chen, Guangping Dai, Timothy G Reese, William J Kostis, Aravinda Thiagalingam, Pal Maurovich-Horvat, Jeremy N Ruskin, Udo Hoffmann, Marcel P Jackowski, David E Sosnovik

**Affiliations:** 1 Athinoula A. Martinos Center For Biomedical Imaging, Boston, MA, USA; 2 Department of Radiology, Massachusetts General Hospital, Harvard Medical School, Boston, MA, USA; 3 Cardiology Division, Massachusetts General Hospital, Harvard Medical School, Boston, MA, USA; 4 Department of Computer Science, Institute of Mathematics and Statistics, University of São Paulo, São Paulo, Brazil; 5 Harvard-MIT Division of Health Sciences and Technology, Cambridge, MA, USA; 6 Athinoula A. Martinos Center For Biomedical Imaging, 149 13th Street, Charlestown, MA, 02129, USA

**Keywords:** Diffusion tensor imaging, Tractography, Myocardium, Remodeling, Heart

## Abstract

**Background:**

The study of myofiber reorganization in the remote zone after myocardial infarction has been performed in 2D. Microstructural reorganization in remodeled hearts, however, can only be fully appreciated by considering myofibers as continuous 3D entities. The aim of this study was therefore to develop a technique for quantitative 3D diffusion CMR tractography of the heart, and to apply this method to quantify fiber architecture in the remote zone of remodeled hearts.

**Methods:**

Diffusion Tensor CMR of normal human, sheep, and rat hearts, as well as infarcted sheep hearts was performed *ex vivo*. Fiber tracts were generated with a fourth-order Runge-Kutta integration technique and classified statistically by the median, mean, maximum, or minimum helix angle (HA) along the tract. An index of tract coherence was derived from the relationship between these HA statistics. Histological validation was performed using phase-contrast microscopy.

**Results:**

In normal hearts, the subendocardial and subepicardial myofibers had a positive and negative HA, respectively, forming a symmetric distribution around the midmyocardium. However, in the remote zone of the infarcted hearts, a significant positive shift in HA was observed. The ratio between negative and positive HA variance was reduced from 0.96 ± 0.16 in normal hearts to 0.22 ± 0.08 in the remote zone of the remodeled hearts (p<0.05). This was confirmed histologically by the reduction of HA in the subepicardium from −52.03° ± 2.94° in normal hearts to −37.48° ± 4.05° in the remote zone of the remodeled hearts (p < 0.05).

**Conclusions:**

A significant reorganization of the 3D fiber continuum is observed in the remote zone of remodeled hearts. The positive (rightward) shift in HA in the remote zone is greatest in the subepicardium, but involves all layers of the myocardium. Tractography-based quantification, performed here for the first time in remodeled hearts, may provide a framework for assessing regional changes in the left ventricle following infarction.

## Background

The microscopic arrangement of myofibers constrains the diffusion of water molecules such that their mobility is not the same in all directions. Diffusion Tensor MR Imaging (DTI) represents the 3D distribution of this mobility as a tensor field. In the heart, water diffuses preferentially along myofibers, allowing fiber orientation to be resolved with diffusion-encoded cardiovascular magnetic resonance (CMR) [1-5]. At each voxel, the principal direction of diffusion is indicated by the primary eigenvector of the tensor. The integration of these eigenvectors into coherent streamlines allows fiber tracts in the heart to be visualized as continuous and functional anatomical entities [6]. Tractography of the heart, however, remains a highly qualitative technique. The purpose of this study was thus to 1) develop a novel set of tools, designed specifically for quantitative tractography in the heart across a range of species, scales, and image resolutions and 2) use these tools to study fiber architecture in the remote zone of remodeled hearts.

Here, we introduce a novel quantitative framework based on the myofiber helix angle (i.e. inclination angle out of the short axis plane of the left ventricle) to characterize 3D fiber anatomy in the heart. We demonstrate that significant changes in the helix angle (HA) are seen in the apical and basal portions of myofibers as they adapt to the shape of the left ventricle (LV). A statistical classification framework based on the HA-encoded streamlines is introduced, and a technique to measure tract coherence in cardiac tractography datasets is described. Using these techniques, we demonstrate that significant changes in myofiber organization occur in the remote zone of an infarct as the heart dilates and remodels. To the best of our knowledge, this is the first description of fiber reorganization in the remodeled LV using quantitative and whole-heart 3D tractography.

## Methods

### Experimental protocol

A total of 20 hearts were studied with full institutional approval: human (n = 5), rat (n = 5), sheep (n = 5), and infarcted sheep (n = 5). Prior to euthanasia, the sheep and rats were injected intravenously with heparin to prevent clot formation in the heart. The excised hearts were perfused and rinsed in normal saline. The human hearts were harvested from organ donors and flown to our institution in a preservative solution on ice. All hearts in the study were perfused-fixed in 4% paraformaldehyde and imaged *ex vivo*. We, and others, have previously shown that fixation does not change fiber orientation in the myocardium [1,2,6-13].

Large anteroseptal infarcts were created percutaneously (3-hour balloon occlusion of the mid left anterior descending coronary artery) in the second group of sheep hearts 3 months prior to euthanasia. The 3-month time point was chosen to ensure that the remodeling process would be fully evolved. Infarction was confirmed visually, as well as through voltage mapping of the left and right ventricles. To evaluate the degree of post-infarct remodeling, volumetric analysis of the LV cavity was performed using the T2-weighted (b=0 s/mm^2^) images for each normal and infarcted sheep heart. Diffusion CMR tractography was used to characterize fiber organization in the lateral wall of each heart. The fiber architecture of each species was compared, and furthermore differences between normal myocardium and the remote zone of remodeled hearts were quantified.

The hearts were immersed in a fluorocarbon-matching medium (Fomblin, Ausimont, NJ) and imaged on a clinical 3T MR scanner (TRIO, Siemens, Erlangen, Germany). DTI was performed using a fat-suppressed single shot spin echo EPI (echo planar imaging) sequence, oriented along the short axis of the LV. Diffusion encoding gradients were placed on either side of the 180-degree refocusing pulse to produce a b-value of 2000 s/mm^2^. Other parameters included voxel-size = 2 × 2 × 2 mm^3^, TR/TE = 8430/96 ms, and a flip angle of 90 degrees. 50-70 slices (without gaps) were acquired in the short axis of the LV to cover the entire heart. Six diffusion-encoding directions were acquired with 24 averages, producing a total scan time of approximately 30 minutes.

DTI of the rat hearts was performed on a 4.7T scanner (Biospec, Bruker, Billerica MA) with a 400 mT/m gradient insert, a tailored solenoid radiofrequency coil, and a diffusion-encoded 3D fat-suppressed spin echo EPI sequence. Image resolution was set to 400 × 400 × 400 μm^3^, TR/TE was 1500/40 ms, 24 diffusion-encoding directions with 2 averages were used, and the b-value used was 1866 s/mm^2^.

### Fiber tracking

Analysis of the data was performed using the dynamic programming language Python in integration with C++ and the Visualization Toolkit (VTK) libraries. For each voxel in the 3D datasets, we computed a dyadic tensor comprising the primary, secondary, and tertiary eigenvectors (ê_1_, ê_2_, and ê_3_) and their associated eigenvalues (λ_1_ > λ_2_ > λ_3_). Fiber bundles were created by integrating the primary eigenvector field into streamlines using a fourth-order Runge-Kutta algorithm with a step length equivalent to one-fourth of the voxel size. A propagation angle (i.e. angle between adjacent streamline vectors) of greater than 35 degrees was used as the single termination criterion. This angle-transition threshold has been used in prior tractographic reconstructions of the heart and brain [14,15].

### Rationale for tractography-based HA classification

**Figure 1:**
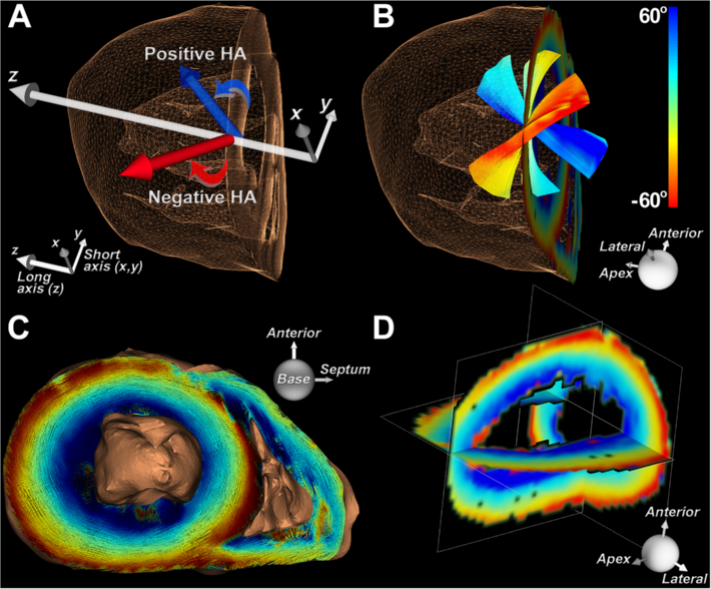
**Representation of fibers in the heart based on their helix angles.** The images shown are of a normal human heart. (**A**) The helix angle (HA) is defined by the inclination of the fiber out of the short axis plane. In the lateral wall of the left ventricle (LV), fibers with a positive HA course towards the antero-apex and those with a negative HA course towards the postero-apex. (**B**) Fiber tracts in the subendocardium, midmyocardium and subepicardium of the lateral wall are shown. Fibers in the subendocardium have a positive HA, those in the subepicardium have a negative HA, and fibers in the midmyocardium are circumferential. (**C**) 3D short-axis view (base to apex) of myofiber tracts color-coded by HA. (**D**) Orthogonal multiplanar view of HA in the left ventricle.

Myofiber HA, the inclination angle that the fiber makes out of the short axis plane of the LV (Figure 1A), is the measure commonly used to describe myofiber architecture [1-5]. Fibers in the subendocardium have a positive HA, those in the midmyocardium are circumferential, while those in the subepicardium have a negative HA (Figure 1B-D). As shown in Figure 1B, HA along a given myofiber can vary substantially. Here, by classifying myofibers as continuous 3D entities, we move beyond voxel-based analysis to a regional evaluation of myocardial microstructure.

**Figure 2:**
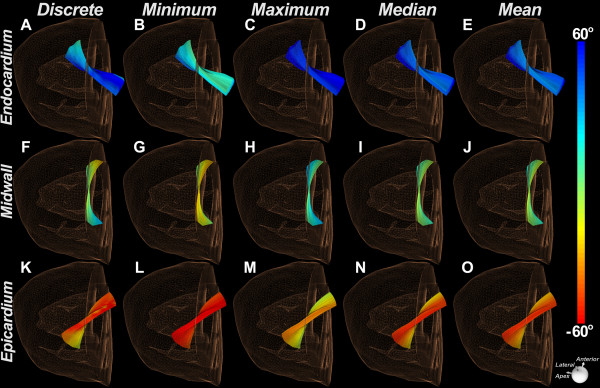
**Myofiber tracts color-coded using the HA-statistical framework.** In the first column (**A**, **F**, **K**) the HA along the tract is classified discretely in each streamline. In all other columns, a single HA is derived for the entire tract based on either the minimum (**B**, **G**, **L**) maximum (**C**, **H**, **M**), median (**D**, **I**, **N**) or mean (**E**, **J**, **O**) HA value along the tract. Endocardial fibers are shown in the first row (**A**-**E**), midmyocardial fibers in the second row (**F**-**J**) and subepicardial fibers in the third row (**K**-**O**). The images are of a normal human heart.

A statistical framework was developed to determine how to optimally describe a continuous myofiber tract in terms of HA. The HA assigned to each tract was defined in one of two ways: 1) a discretized approach in which voxel-based HA values were assigned along the tract, and 2) a statistical approach in which a single HA was computed and assigned to the entire tract based on HA statistics along the tract (Figure 2). The HA value assigned to the tract under this latter technique could be defined by the minimum, maximum, median, or mean HA along the tract (Figure 2). This statistical HA classification framework was used to derive a measure of tract coherence in cardiac datasets, the tractographic coherence index (TCI).

### Derivation of the tractographic coherence index (TCI)

**Figure 3:**
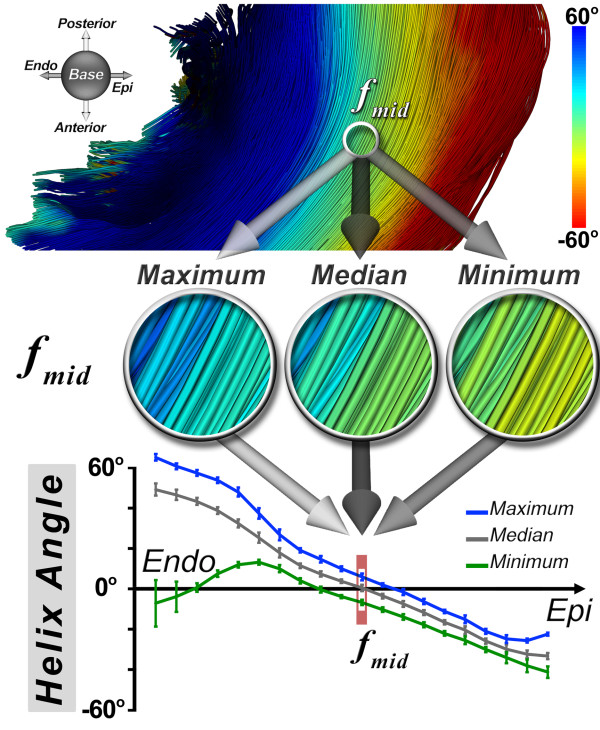
**Derivation of the tractographic coherence index (TCI).** The determination of the TCI is based on the maximum, median, and minimum HA classifications of myofibers within a ROI. This classification is depicted for myofibers in the midmyocardium (*f*_*mid*_). The maximum, median, and minimum HA curves are derived by averaging each HA classification successively in the base-apex and anterior-posterior directions of the ROI at each voxel. This creates the 3 transmural HA curves. The generated curves describe the transmural gradients in the maximum, median, and minimum myofiber HA, and are derived with fiber length limited to πR or the ROI. In the midmyocardium and subepicardium, the curves lie close to each other, consistent with highly coherent tracts, whereas in the subendocardium, tract coherence is lost due to the effect of the papillary muscles and trabeculations.

The determination of the TCI is based on the maximum, median, and minimum HA classifications of myofibers within a ROI, the dimensions of which were 1460 ± 370 mm^3^ in the human hearts, 590 ± 140 mm^3^ in the sheep hearts and 86 ± 17 mm^3^ in the rat hearts. An example of this classification is shown at the midmyocardium (f_mid_) in Figure 3. To obtain the maximum, median, and minimum HA curves, a two-step process is performed independently for each statistical HA classification. First, the average of each HA classification is calculated in the base to apex direction of the ROI at each voxel. This creates a two-dimensional average HA map for each classification, which is again averaged in the anterior-posterior direction, producing 3 transmural HA curves. The generated curves describe the transmural gradients in the maximum, median, and minimum myofiber HA. This process is performed, firstly, by considering fiber length limited to πR and secondly to the dimensions of the ROI. The TCI is then defined as the reciprocal of the normalized quadratic error of the differences in the minimum (HA_min_) and maximum (HA_max_) from the median (HA_med_) HA profiles across the myocardium and is given by:

$$\text{TCI}=\left[\frac{\int_{\text{ENDO}}^{\text{EPI}}{\left({\text{HA}}_{\mathrm{med}}\left(x\right)\right)}^{2}dx}{\int_{\text{ENDO}}^{\text{EPI}}{\left({\text{HA}}_{\max}\left(x\right)-{\text{HA}}_{\mathrm{med}}\left(x\right)\right)}^{2}dx+\int_{\text{ENDO}}^{\text{EPI}}{\left({\text{HA}}_{\min}\left(x\right)-{\text{HA}}_{\mathrm{med}}\left(x\right)\right)}^{2}dx}\right]$$

The TCI values were calculated for normal and remodeled myocardium across different fiber lengths and image resolutions. The goals of this index are 1) to assess the quality of tractographic datasets and 2) to determine whether changes in myocardial microstructure following infarction occur in a coherent fashion.

### Tissue sectioning and microscopy

Histological validation of the DTI-derived HA values was performed using phase-contrast microscopy. Tissue specimens from the lateral wall of each sheep heart (n=10) were embedded in paraffin and sectioned into 5-μm-thick slices tangential to the epicardial surface of the heart. Sections were obtained at 6 equidistant transmural levels from epicardium to endocardium. Phase-contrast microscopy was performed on the sections at 100 × magnification using an Olympus IX51 microscope (Center Valley, PA USA). Images were acquired under the bright field filter, natural contrast, and optimal exposure settings.

To quantify the HA from the histologic sections, microscopy images were analyzed through a Hessian matrix-based algorithm [16] allowing the fibers’ local orientations and interstitial spaces to be identified from local intensity information. Hence, regions of coherent orientation were identified as locations depicting local extrema of the principal intensity curvatures. As a result of this process, line segments representing fibers and interstitial spaces were extracted and subsequently eroded to yield segments of single pixel thickness. The HA was then computed to quantify the angle between each connected segment and the horizontal axis. A mean value of HA was computed for all segments resulting in an average HA for each transmural section.

## Results

### Statistical HA classification of normal hearts

**Figure 4:**
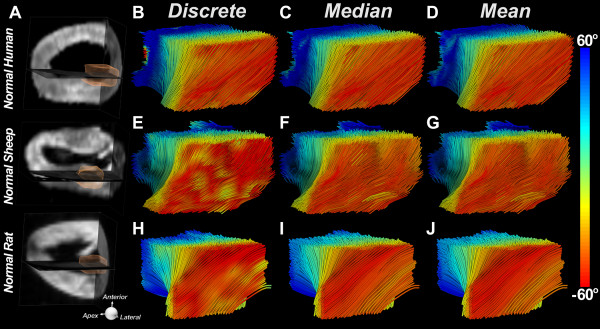
**HA classification of fiber tracts limited in length to a ROI placed in the lateral wall of the LV (A).** Myofiber tracts in normal human (**B**-**D**), sheep (**E**-**G**), and rat (**H**-**J**) hearts have been reconstructed. The hearts are being viewed from the epicardial surface (red fibers) of their lateral walls and their fiber organization is well visualized with the discrete (**B**, **E**, **H**) median (**C**, **F**, **I**) and mean (**D**, **G**, **J**) HA classifications. The transmural variation in HA is highly similar in all three species.

**Figure 5:**
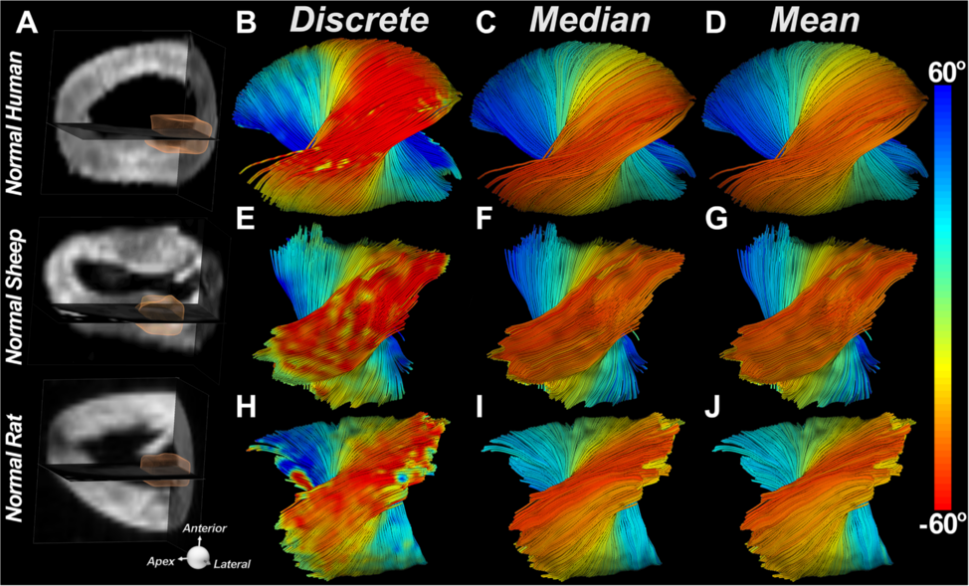
**HA classification of fiber tracts passing through a ROI placed in the lateral wall of the LV (A).** The tracts have been limited in length to half the LV circumference (πR). Tracts in the lateral wall of normal human (**B**-**D**) normal sheep (**E**-**G**) and normal rat hearts (**H**-**J**) are shown. The discrete HA maps (**B**, **E**, **H**) show that HA is highest in the midventricular portion of a given myofiber and decreases as the fiber approaches either the base or the apex. Only a slight reduction in HA range is produced by median HA classification (**C**, **F**, **I**). The reduction in HA range with mean HA classification (**D**, **G**, **J**) is slightly larger.

Fibers limited to a transmural region of interest (ROI) placed in the lateral wall at the mid-ventricular level of normal human, sheep and rat hearts are shown in Figure 4. The same three hearts are shown in Figure 5, with fiber length limited to half the LV circumference (πR). Discrete HA encoding (Figure 5B, 5E, 5H) reveals that the HA is steeper in the mid-ventricular region of myofibers and flattens (more circumferential) as the fiber approaches the apex and base of the LV. Fiber organization in the three species is extremely similar, with the endocardial and epicardial fibers crossing over each other with an angle of 100-120 degrees. Classification of myofibers by their median HA, rather than by the discrete HA, resulted in only a small reduction in the transmural HA range.

### Architectural reorganization in remote zone

**Figure 6:**
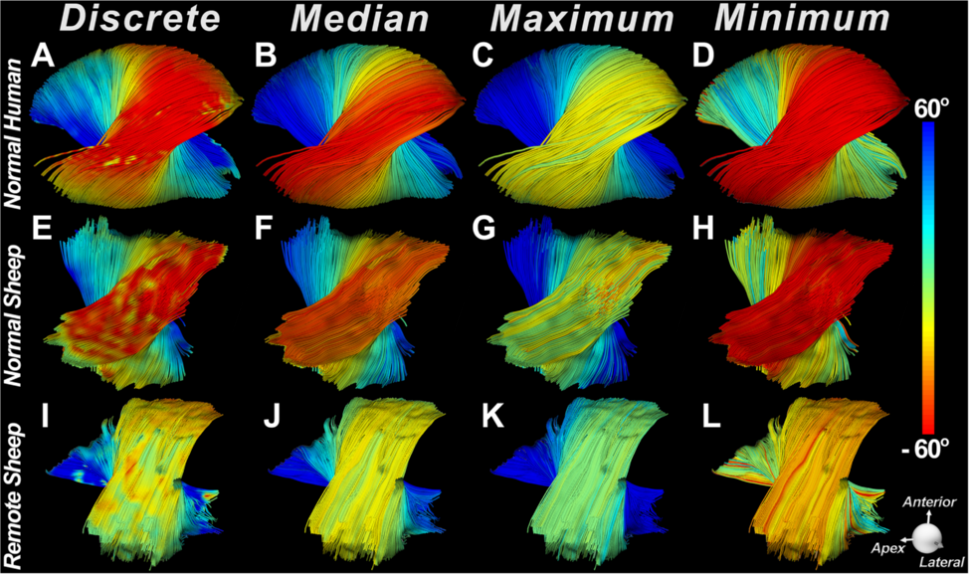
**HA classification of fiber tracts in normal and infarcted sheep hearts.** Fiber tracts in the lateral wall of (**A**-**D**) a normal human heart and (**E**-**H**) a normal sheep heart are shown. (**I**-**L**) Fiber tracts in the lateral wall (remote zone) of a sheep heart with a large anteroseptal infarct. The hearts are being viewed from their epicardial surface. In the normal human and sheep hearts (**A**, **E**) the subepicardial fibers have a highly negative HA (red) and form a symmetric array of crossing helices with the subendocardial fibers. (**I**) The subepicardial fibers in the remote zone of the infarcted sheep heart have undergone a rightward rotation and no longer have a highly negative HA. Compared to the subepicardial fibers in the normal sheep heart (**E***vs*. **I**), the subepicardial fibers in the remote zone have a more circumferential orientation (yellow rather than red). The rightward shift in the remote zone is well seen with median HA classification (**F***vs*. **J**) as well as with the minimum HA classification (**H***vs*. **L**).

Tractography was performed in the lateral wall of normal human, normal sheep, and infarcted sheep hearts. End-systolic volume on the T2-weighted images was, on average, 111% higher in the infarcted than the normal sheep hearts, consistent with extensive remodeling [17]. Fiber tracts in the lateral wall of a normal human, normal sheep, and infarcted sheep heart (remote zone) are shown in Figure 6. No substantial differences can be seen between the discrete, median, and minimum HA classifications in normal human and sheep hearts with minor variation in HA along most of their fiber length. Fiber organization in the lateral wall (remote zone) of the infarcted sheep hearts, however, differs significantly from that in normal human and sheep hearts. The subepicardial fibers in the remote zone are more circumferential (yellow rather than red) than those in the normal hearts, and have lost a significant portion of their obliquity. This fiber reorganization between normal and infarcted sheep hearts is manifested by a positive HA shift in the discrete, median, maximum, and minimum HA classifications.

**Figure 7:**
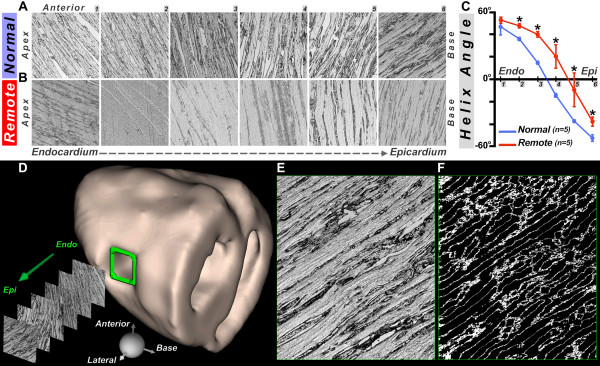
**Phase contrast microscopy of myofiber architecture.** Comparison of histological sections at 6 transmural depths in the lateral wall (1-6, from endocardium to epicardium) of a normal sheep heart (**A**) and the remote zone (**B**) of an infarcted sheep heart confirms a rightward shift of fibers in the remote zone**.** (**C**) Transmural gradient in HA calculated from the histological sections. The zero-crossing of the curve in the remote zone is significantly shifted towards the epicardium and thus the HA becomes more positive (normal vs. remote: * = p < 0.05). (**D**) Surface rendering of a normal heart depicting the location (green rectangle) of tissue blocks removed from the lateral LV wall for phase contrast microscopy. Sectioning was performed perpendicular to the normal of the epicardial surface of the tissue block. The coordinate system used to calculate fiber HA was identical to that shown for MR-tractography in Figure 1A. (**E**) Histological section at the subepicardial level and (**F**) corresponding ridges after Hessian-based analysis of local intensity structure.

The positive (rightward) shift in fiber architecture in the remote zone was confirmed histologically (Figure 7). In the normal hearts (Figure 7A), fiber HA was fairly symmetric around the midmyocardium, whereas in the remote zone of the infarcted hearts (Figure 7B), a strong rightward shift was observed. A plot of average histological HA in the lateral wall of the normal and infarcted sheep hearts is shown in Figure 7C. Histological HA (mean ± SD) in the most epicardial level (level 6) was −52.03 ± 2.94 degrees in the normal hearts and −37.48 ± 4.05 degrees in the remote zone of the infarcted hearts (p < 0.05, Mann-Whitney). The differences in the histological HA shown in Figure 7C were significant (p < 0.05) at all transmural levels except for the most subendocardial level (level 1).

**Figure 8:**
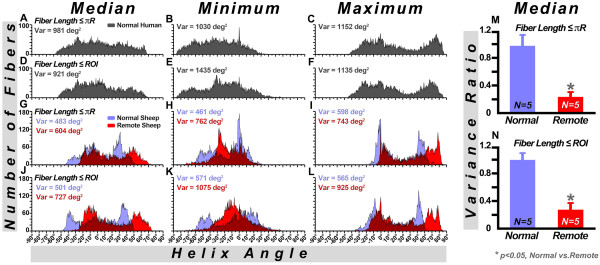
**HA histograms of normal human, normal sheep and infarcted sheep hearts.** HA Histograms in a normal human heart with fiber length limited to πR (**A**-**C**) and the dimensions of a small ROI (**D**-**F**). HA histograms of a normal and an infarcted (remote zone) sheep heart have been superimposed (**G**-**L**). The histogram of the normal heart is in mauve, the remote zone is in bright red, and areas where the histograms overlap are in dark red. The histograms in the remote zone are significantly shifted to the right, both with fiber length limited to πR (**G**-**I**) and the ROI (**J**-**L**). The variance ratio (i.e. the ratio between the negative and positive variance in the median HA histogram) is significantly reduced in the remote zone, consistent with the rightward HA shift, both when fiber length is limited to πR (**M**) and to the ROI (**N**).

Histograms of myofiber HA in the lateral wall are shown in Figure 8. The fibers have been classified by their median, minimum, or maximum HA values, and limited in length to πR or to a transmural ROI. In the human hearts, fibers ranged from −60 to +60 degrees, and were symmetrically arranged around zero with little skewness (Figure 8A, 8D). A similar symmetric arrangement around zero was seen in the normal sheep hearts, but with a slightly lower overall range of −50 to +50 degrees (Figure 8G, 8J). Histograms of fiber architecture in the remote zone, however, showed a marked positive shift in myofiber HA. This rightward shift in the remote zone was quantified by calculating the HA variance ratio (i.e. ratio between the variance of the negative HA values and that of the positive HA values in the median HA histogram). With fiber length set to πR (Figure 8M), the HA variance ratio (mean ± SD) was 0.96 ± 0.16 in the normal sheep hearts versus 0.22 ± 0.08 in the remote zone of the infarcted sheep hearts (p < 0.05, Mann-Whitney). With fiber length constrained to the ROI (Figure 8N), these ratios were 0.99 ± 0.10, and 0.27 ± 0.10, respectively (p < 0.05, Mann-Whitney).

### TCI in normal and infarcted hearts

**Figure 9:**
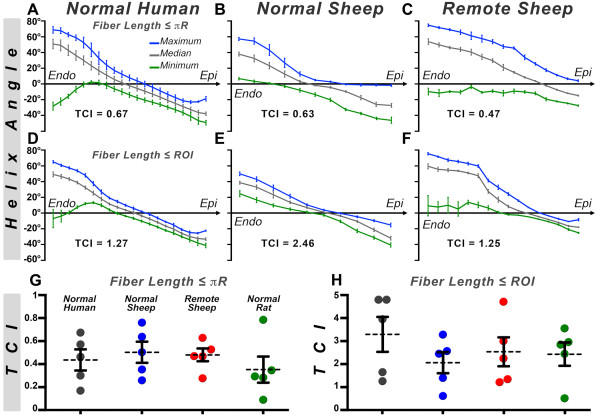
**TCI values in normal and infarcted hearts.** (**A**-**F**) HA curves in normal human, normal sheep, and the remote zone of infarcted sheep hearts. In the normal hearts, the median HA curve (gray) has a symmetric arrangement around its zero-crossing in the midmyocardium. In the remote zone, all three HA curves are markedly shifted to the right (more positive HA). (**G-H**) TCI values in the lateral wall of normal human, sheep, and rat hearts, and the remote zone of infarcted sheep hearts. The TCI remains high in the remote zone, confirming the reorganization of myofiber architecture.

TCI plots in the lateral wall of normal human, normal sheep, and the remote zone of infarcted sheep hearts are shown in Figure 9. In the normal hearts, the median HA curve (gray) encloses similar areas above (positive HA) and below (negative HA) the horizontal axis, and the zero-crossing of the curve occurs close to the midmyocardium. In the remote zone of the infarcted hearts, however, the median HA curves (gray, Figure 9C, 9F) undergo a transmural positive shift in HA. Hence, the zero-crossing is displaced towards the subepicardium, which results in a loss of transmural balance between positive and negative HA fibers. The reorientation of myofibers in the remote zone was manifested by large rightward shifts in the minimum and maximum HA curves as well. The reorganized myofibers in the remote zone remain highly coherent and are not characterized by abrupt deviations or dispersions in HA.

Average TCI values in the lateral wall of human, sheep, and rat hearts are shown in Figure 9G, 9H. When fiber length was set to πR, TCI values (mean ± SD) were 0.44 ± 0.20, 0.50 ± 0.20, and 0.35 ± 0.26 in the normal human, sheep, and rat hearts, respectively. The TCI value in the remote zone of the infarcted hearts was 0.48 ± 0.13. When fiber length was limited to a small ROI, TCI values were 3.30 ± 1.72, 2.07 ± 1.06, and 2.44 ± 1.16 in the normal human, sheep, and rat hearts, respectively. The TCI value in the remote zone of the infarcted hearts was 2.55 ± 1.44. No significant differences in TCI values were seen between normal myocardium and the remote zone of infarcted hearts, consistent with the uniform quality of the datasets and the coherent nature of the fiber reorganization in the remodeled hearts.

As shown in Figure 9, TCI values between species are similar and should be greater than 0.1 when fiber length is equal to πR, and even higher with shorter tract lengths. A slight degree of HA incoherence along myofiber tracts is normal because the HA along the fiber fluctuates at the apex and base. In addition, the orientation of the HA in the papillary muscles and trabeculations can be highly variable. However, TCI values lower than 0.1 reflect large variations in HA and may suggest either noisy data or myofiber disorganization due to disease. TCI values in the remote zone were similar to normal myocardium, indicating that the rightward reorientation in fiber architecture was coherent and occurring with little dispersion in HA along the entire length of the fiber.

## Discussion

The application of tractography in the heart has been limited and largely qualitative [4,9,14,18-21]. Here, we introduce a statistical framework to quantify 3D tractography of the myocardium. We demonstrate that fiber HA can vary significantly along a myofiber tract, and that fiber classification by median HA provides a robust measure of 3D myofiber architecture. Using this quantitative technique, we demonstrate that fiber architecture in the remote zone of an infarct differs significantly from fiber architecture in normal myocardium.

A symmetrical distribution of fibers with positive (subendocardial) and negative (subepicardial) helix angles has been reported in most species [1-5,8,9,21-25]. This symmetrical pattern was seen in the normal sheep hearts, but was lost in the remote zone of infarcted hearts where a significant rightward shift in fiber orientation was observed. This shift could be detected by visual inspection of the fiber tracts, was quantified using histograms of myofiber HA, and was confirmed by histology. The TCI values in the remote myocardium, however, did not differ from those obtained at the identical location in the lateral wall of normal myocardium. This indicates that the rightward shift in fiber orientation within the remote zone occurred in a coherent fashion along the length of the myofiber.

Myofiber organization in the remote zone after myocardial infarction has not been extensively studied. A 2D DTI study in pigs documented an increase in left-handed fibers in the remote zone of anterior infarcts [5]. Two studies have used *in vivo* 2D DTI to evaluate fiber orientation in the remote zone of patients with myocardial infarcts [26,27]. In both, a significant reduction in left-handed fibers was documented in the remote zone. These *in vivo* 2D human studies are thus in strong agreement with the results of our study, which also show a reduction in left-handed fibers in the remote zone. Here, we introduce a 3D tractographic approach to complement and extend the observations made in these prior 2D DTI studies [26,27]. This 3D integrative technique offers the advantage of considering myofibers as continuous 3D entities when quantifying the architectural reorganization that accompanies myocardial infarction.

The cause of the fiber reorganization in the remote zone of infarcted hearts will require further study. We hypothesize that dilation of the LV imposes the greatest microstructural stress on the fibers closest to its outer surface, where radial distance from the center of the LV is highest. These subepicardial fibers must elongate further to adapt to the new outer circumference of the dilated LV. Without sufficient elongation, the subepicardial fibers are forced to lose obliquity and assume a more circumferential orientation. The rightward shift was greatest in the subepicardium and midmyocardium, but was observed in all layers of the remote zone in our study (Figure 7). Regardless of the nature of this remodeling process, the rightward reorientation of myofibers in the remote zone may contribute to the progressive loss of function over time. Future work will focus on the integration of 3D tractography and myocardial strain, which will likely provide powerful insights into the relationship between myocardial structure and function.

## Conclusions

In this study, we provide a quantitative framework that systematically classifies continuous fiber tracts in the myocardium. Median, minimum and maximum HA are used in the current scheme, however our approach supports the use of statistical indices including HA variance and momentum analysis. Further investigation will determine the level of incremental value derived from the use of these and other indices. The strength of the developed approach is demonstrated here by its ability to detect changes in myofiber organization in the remote zone of remodeled hearts. The method we describe is robust, and has the potential to promote tractography of the myocardium from a qualitative technique to a quantitative one. This study, thus, constitutes an important step towards the standardization, broader use, and greater utility of diffusion CMR tractography in the heart. Furthermore, this quantitative framework may enhance the understanding of the underlying myocardial architectural properties involved in the post-myocardial infarction remodeling process.

## Abbreviations

DTI: Diffusion Tensor CMR; CMR: Cardiovascular Magnetic Resonance; LV: Left Ventricle; HA: Helix Angle; EPI: Echo Planar Imaging; TR: Repetition Time; TE: Echo Time; TCI: Tractographic Coherence Index; ROI: Region of Interest.

## Competing interests

David E Sosnovik: Research support from and consultant for Siemens Medical Solutions Inc.

## Authors’ contributions

CM: Conception and design of the study, acquisition and analysis of the data, development of the analysis software, generation of the figures, writing of the manuscript. SH: Conception and design of the study, acquisition and analysis of the data, editing of the manuscript. HHC: Conception and design of the study, acquisition and analysis of the data, editing of the manuscript. GD: Conception and design of the study, development of the acquisition pulse sequence, acquisition and analysis of the data, editing of the manuscript. TGR: Conception and design of the study, development of the acquisition pulse sequence, acquisition and analysis of the data, editing of the manuscript. WJK: Design of study analysis, Development of analysis algorithms, analysis of the data, writing and editing of the manuscript. AT: Conception and design of the study, acquisition and analysis of the data, editing of the manuscript. PM-H: Conception and design of the study, acquisition and analysis of the data, editing of the manuscript. JNR: Conception and design of the study, editing of the manuscript. UH: Conception and design of the study, editing of the manuscript. MPJ: Conception and design of the study, analysis of the data, development of the analysis software, generation of the figures, writing of the manuscript. DE S: Conception and design of the study, acquisition and analysis of the data, development of the analysis software, generation of the figures, writing of the manuscript, funding of the study, overall supervision of the study. All authors read and approved the final manuscript.

## References

[B1] Hsu EW Muzikant AL Matulevicius SA Penland RC Henriquez CS Magnetic resonance myocardial fiber-orientation mapping with direct histological correlation Am J Physiol 1998 274 H1627 H1634 9612373 10.1152/ajpheart.1998.274.5.H1627

[B2] Scollan DF Holmes A Winslow R Forder J Histological validation of myocardial microstructure obtained from diffusion tensor magnetic resonance imaging Am J Physiol 1998 275 H2308 H2318 9843833 10.1152/ajpheart.1998.275.6.H2308

[B3] Chen J Liu W Zhang H Lacy L Yang X Song SK Wickline SA Yu X Regional ventricular wall thickening reflects changes in cardiac fiber and sheet structure during contraction: quantification with diffusion tensor MRI Am J Physiol Heart Circ Physiol 2005 289 H1898 H1907 10.1152/ajpheart.00041.2005 16219812

[B4] Strijkers GJ Bouts A Blankesteijn WM Peeters TH Vilanova A van Prooijen MC Sanders HM Heijman E Nicolay K Diffusion tensor imaging of left ventricular remodeling in response to myocardial infarction in the mouse NMR Biomed 2009 22 182 190 10.1002/nbm.1299 18780284

[B5] Wu EX Wu Y Nicholls JM Wang J Liao S Zhu S Lau CP Tse HF MR diffusion tensor imaging study of postinfarct myocardium structural remodeling in a porcine model Magn Reson Med 2007 58 687 695 10.1002/mrm.21350 17899595

[B6] Sosnovik DE Wang R Dai G Reese TG Wedeen VJ Diffusion MR tractography of the heart J Cardiovasc Magn Reson 2009 11 47 10.1186/1532-429X-11-47 19912654 PMC2781805

[B7] Holmes AA Scollan DF Winslow RL Direct histological validation of diffusion tensor MRI in formaldehyde-fixed myocardium Magn Reson Med 2000 44 157 161 10.1002/1522-2594(200007)44:1<157::AID-MRM22>3.0.CO;2-F 10893534

[B8] Tseng WY Reese TG Weisskoff RM Brady TJ Wedeen VJ Myocardial fiber shortening in humans: initial results of MR imaging Radiology 2000 216 128 139 10887238 10.1148/radiology.216.1.r00jn39128

[B9] Helm P Beg MF Miller MI Winslow RL Measuring and mapping cardiac fiber and laminar architecture using diffusion tensor MR imaging Ann N Y Acad Sci 2005 1047 296 307 10.1196/annals.1341.026 16093505

[B10] LeGrice IJ Smaill BH Chai LZ Edgar SG Gavin JB Hunter PJ Laminar structure of the heart: ventricular myocyte arrangement and connective tissue architecture in the dog Am J Physiol 1995 269 H571 H582 7653621 10.1152/ajpheart.1995.269.2.H571

[B11] Scollan DF Holmes A Zhang J Winslow RL Reconstruction of cardiac ventricular geometry and fiber orientation using magnetic resonance imaging Ann Biomed Eng 2000 28 934 944 11144678 10.1114/1.1312188 PMC1473035

[B12] Tseng WY Wedeen VJ Reese TG Smith RN Halpern EF Diffusion tensor MRI of myocardial fibers and sheets: correspondence with visible cut-face texture J Magn Reson Imaging 2003 17 31 42 10.1002/jmri.10223 12500272

[B13] Helm PA Tseng HJ Younes L McVeigh ER Winslow RL Ex vivo 3D diffusion tensor imaging and quantification of cardiac laminar structure Magn Reson Med 2005 54 850 859 10.1002/mrm.20622 16149057 PMC2396270

[B14] Sosnovik DE Wang R Dai G Wang T Aikawa E Novikov M Rosenzweig A Gilbert RJ Wedeen VJ Diffusion spectrum MRI tractography reveals the presence of a complex network of residual myofibers in infarcted myocardium Circ Cardiovasc Imaging 2009 2 206 212 10.1161/CIRCIMAGING.108.815050 19808594 PMC2760045

[B15] Mori S van Zijl PC Fiber tracking: principles and strategies - a technical review NMR Biomed 2002 15 468 480 10.1002/nbm.781 12489096

[B16] Eberly D Gardner R Morse B Pizer S Scharlach C Ridges for image analysis Journal of Mathematical Imaging and Vision 1994 4 353 373 10.1007/BF01262402

[B17] Hung J Guerrero JL Handschumacher MD Supple G Sullivan S Levine RA Reverse ventricular remodeling reduces ischemic mitral regurgitation: echo-guided device application in the beating heart Circulation 2002 106 2594 2600 10.1161/01.CIR.0000038363.83133.6D 12427657

[B18] Rohmer D Sitek A Gullberg GT Reconstruction and visualization of fiber and laminar structure in the normal human heart from ex vivo diffusion tensor magnetic resonance imaging (DTMRI) data Invest Radiol 2007 42 777 789 10.1097/RLI.0b013e3181238330 18030201

[B19] Toussaint N Sermesant M Stoeck CT Kozerke S Batchelor PG In vivo human 3D cardiac fibre architecture: reconstruction using curvilinear interpolation of diffusion tensor images Med Image Comput Comput Assist Interv 2010 13 418 425 20879258 10.1007/978-3-642-15705-9_51

[B20] Smerup M Nielsen E Agger P Frandsen J Vestergaard-Poulsen P Andersen J Nyengaard J Pedersen M Ringgaard S Hjortdal V The three-dimensional arrangement of the myocytes aggregated together within the mammalian ventricular myocardium Anat Rec (Hoboken) 2009 292 1 11 10.1002/ar.20798 19051244

[B21] Ennis DB Nguyen TC Riboh JC Wigstrom L Harrington KB Daughters GT Ingels NB Miller DC Myofiber angle distributions in the ovine left ventricle do not conform to computationally optimized predictions J Biomech 2008 41 3219 3224 10.1016/j.jbiomech.2008.08.007 18805536 PMC2612586

[B22] Geerts L Bovendeerd P Nicolay K Arts T Characterization of the normal cardiac myofiber field in goat measured with MR-diffusion tensor imaging Am J Physiol Heart Circ Physiol 2002 283 H139 H145 12063284 10.1152/ajpheart.00968.2001

[B23] Streeter DD Jr Spotnitz HM Patel DP Ross J Jr Sonnenblick EH Fiber orientation in the canine left ventricle during diastole and systole Circ Res 1969 24 339 347 10.1161/01.RES.24.3.339 5766515

[B24] Harrington KB Rodriguez F Cheng A Langer F Ashikaga H Daughters GT Criscione JC Ingels NB Miller DC Direct measurement of transmural laminar architecture in the anterolateral wall of the ovine left ventricle: new implications for wall thickening mechanics Am J Physiol Heart Circ Physiol 2005 288 H1324 H1330 15550521 10.1152/ajpheart.00813.2004 PMC2822837

[B25] Chen J Song SK Liu W McLean M Allen JS Tan J Wickline SA Yu X Remodeling of cardiac fiber structure after infarction in rats quantified with diffusion tensor MRI Am J Physiol Heart Circ Physiol 2003 285 H946 H954 12763752 10.1152/ajpheart.00889.2002

[B26] Wu MT Tseng WY Su MY Liu CP Chiou KR Wedeen VJ Reese TG Yang CF Diffusion tensor magnetic resonance imaging mapping the fiber architecture remodeling in human myocardium after infarction: correlation with viability and wall motion Circulation 2006 114 1036 1045 10.1161/CIRCULATIONAHA.105.545863 16940196

[B27] Wu MT Su MY Huang YL Chiou KR Yang P Pan HB Reese TG Wedeen VJ Tseng WY Sequential changes of myocardial microstructure in patients postmyocardial infarction by diffusion-tensor cardiac MR: correlation with left ventricular structure and function Circ Cardiovasc Imaging 2009 2 32 40 10.1161/CIRCIMAGING.108.778902 19808562

